# A landscape-scale field survey demonstrates the role of wheat volunteers as a local and diversified source of leaf rust inoculum

**DOI:** 10.1038/s41598-023-47499-6

**Published:** 2023-11-21

**Authors:** A.-L. Boixel, H. Goyeau, J. Berder, J. Moinard, F. Suffert, S. Soubeyrand, I. Sache, T. Vidal

**Affiliations:** 1 Université Paris-Saclay, INRAE, UR BIOGER, 91123 Palaiseau, France; 2 DRAAF Midi-Pyrénées, 31074 Toulouse, France; 3 INRAE, BioSP, 84914 Avignon, France; 4 AgroParisTech, 91123 Palaiseau, France

**Keywords:** Ecology, Plant sciences

## Abstract

Deploying disease-resistant cultivars is one of the most effective control strategies to manage crop diseases such as wheat leaf rust, caused by *Puccinia triticina*. After harvest, this biotrophic fungal pathogen can survive on wheat volunteers present at landscape scale and constitute a local source of primary inoculum for the next cropping season. In this study, we characterised the diversity of *P. triticina* populations surveyed on wheat volunteer seedlings for six consecutive years (2007–2012) at the landscape scale. A total of 642 leaf rust samples classified in 52 virulence profiles (pathotypes) were collected within a fixed 5-km radius. The pathotype composition (identity and abundance) of field-collected populations was analyzed according to the distance between the surveyed wheat plots and to the cultivars of origin of isolates. Our study emphasised the high diversity of *P. triticina* populations on wheat volunteers at the landscape scale. We observed an impact of cultivar of origin on pathogen population composition. Levels of population diversity differed between cultivars and their deployment in the study area. Our results suggest that wheat volunteers could provide a significant though highly variable contribution to the composition of primary inoculum and subsequent initiation of leaf rust epidemics.

## Introduction

Improving crop yield stability is crucial in the context of global population rise and climate change^1^. Crop diseases routinely lead to large yield losses thus threatening food security^2^. An efficient strategy to manage these diseases is, rather than using pesticides causing environmental impacts, deploying cultivars (cultivated varieties) that carry different sources of resistance to plant pathogens^3^.

Yield losses generally result from symptoms that are responsible for disease progression within fields during the cropping season. However, this stage of expansion of pathogen populations represents only part of its multiannual dynamics. Pathogen population size is considerably reduced during the period between cropping seasons and exchange of inoculum through spore fluxes between adjacent field plots can play an important role. This ‘off-season survival’ phase on their reservoir host populations remains largely unexplored due to its limited impacts on yield, but might have large consequences in terms of pathogen population and spatiotemporal disease dynamics^4^. During this phase, the local pathogen population might get extinct or decline to a level to which local adaptation acquired during the previous cropping season might be wiped out. In this case, crops might be recolonized by an inoculum from distant origin, notably for wind long-distance dispersed pathogens^5^ (e.g. 500 to 2000 km in the case of rusts). Alternatively, if part of the local population is maintained, this bottleneck (host harvest in the case of biotrophic pathogens that require living plant tissue to survive and complete their life cycle) could have large consequences on the overall pathogen diversity, for instance through genetic drift or through the elimination of rare adaptive mutations^3^.

Crop pathogen species can have different strategies to survive unfavorable periods. Some generalist pathogens such as *Colletotrichum* spp. (cause of anthracnose disease in yam) can maintain on wild plants^6^ considered to be secondary or alternative hosts. Other crop pathogen species are more specific. This is the case of wheat leaf rust^7^ (caused by *Puccinia triticina* Eriks., *Pt*) for which the possibility to infect wild grasses is considered highly limited^8^. In some cases, mainly in the cycle of several rust species, switching from asexual to sexual reproduction regime can also contribute to survival during an unfavorable period. In the case of *Pt*, sexual reproduction occurs on *Thalictrum* spp. and *Isopyrum* spp.^9^ (alternate hosts, pycnial-aecial spore stages) but this stage is considered non-significant for leaf rust epidemiology in Western Europe^10^.

The pathogen populations of *Pt* can maintain on wheat volunteers, i.e. wheat plants grown spontaneously after harvest from seeds fallen during harvest. In the case of biotrophic pathogens, wheat volunteers provide a living support during the period between harvest and the emergence of the new following crop. They constitute the summer “green bridge” for the disease (survival of the fungus during the intercrop season) for subsequent infection of wheat plants during the following growing season^11,12^. Infected wheat volunteers could thus be an important source of primary inoculum contributing to the initiation of the next epidemic on the autumn-planted winter-wheat^13^.

Primary inoculum can be issued from more or less distant sources, but local sources, such as infected volunteers, are expected to provide higher amounts of spores. This is due to classical dispersal patterns of windborne diseases such as rusts^14^, for example with most wheat rust spores being dispersed less than 100 m away from the source^15^. Local inoculum is also expected to be more adapted to local environment for example to meteorological conditions^16^ and to cultivars grown locally that filter pathogen populations through host resistance^17^. Local sources of inoculum and their connectivity^18^ could therefore play a critical role in plant pathogen dynamics (colonization, establishment, extinction of populations^19^) and adaptation to host resistances deployed at landscape scale modulated through cultivar cropping.

Soubeyrand et al.^20^ studied temporal continuities and discontinuities in the composition of *Pt* populations at pluri-annual scale, considering both cultivated wheat plants and volunteers. They observed that discontinuities were not systematically associated with interepidemic periods, suggesting that volunteers could contribute significantly to the local persistence of *Pt* populations between cropping seasons. The authors focused on the temporal dimension (disruptions and continuations at successive sampling dates in two sentinel plots) when comparing populations but they did not account for spatial distribution of wheat volunteers in the landscape.

Volunteer populations, which are usually considered as weeds, can be quite heterogeneous, both in time and space. Thus, they can display a highly fragmented spatial distribution^21^ (patchiness, asynchrony) even in a given plot (from one set of plants to a fully-covered field). However, they have the same genetic background as previously cultivated wheat plants in a given plot, which can strongly affect the composition and size of pathogen populations^22^ by acting as a filter^23,24^. The characteristics of the primary inoculum constituted by these populations on volunteers, diversified in terms of strain composition, fitness and spatial distribution, could strongly impact in turn the course of epidemics and how they unfold^25,26^.

Pathogen population dynamics across scales has been pointed out as a critical analysis item^27^ and studied in particular in natural systems^28^ including, in a lesser number of cases, during the off-season^4^. This has been the case for a long-term ecological study on the *Plantago lanceolata*–*Podosphaera plantaginis* pathosystem in the Åland Islands in south-west Finland^26^ (21 years). In order to better understand plant disease epidemics, it is essential to compare pathogen population composition and dynamics at different time (including during off-season survival) and space (over a wheat production basin in the case of *Pt*). This is particularly important in agroecoystems in which the hosts tend to be more genetically uniform^29^. Despite intense interest, direct tests of these ideas in agricultural systems remain scarce.

In this study, we present a landscape-scale field study conducted over six years to test whether cultivar deployment and distance between fields may impact the diversity (in terms of strain composition i.e. identity and abundance) of *Pt* populations recovered from wheat volunteers during the interseason.

## Material and methods

Leaf rust was surveyed for the presence/absence of infection on wheat volunteers for six consecutive years (2007–2012) in a main area of winter wheat production in southwestern France (the Lomagne agricultural area). Samples of wheat leaves from plants infected with the leaf rust fungus were collected within a fixed 5-km area of radius centered around a fixed plot which remained unchanged along the study period. The studied area, without significant urbanized areas but small villages and isolated farms, is made of a mosaic of 911 plots ranging from 3 to 15 ha. Samplings were carried out in the autumn, when previous wheat plots with volunteers (year n-1) co-existed with newly-sown wheat plots (year n). Each year, information was collected concerning the location of wheat plots in the area and the identity of the cultivars sown in these plots. The diversity of isolates with respect to their virulence profiles at the plot scale was characterised and analysed according to the distance between the surveyed wheat plots and the cultivars of origin of isolates.

A total of 642 samples—up to 16 samples per plot, and from between 19 to 25 plots each year—were collected, recovered and pathotyped (Supplementary Material). Sampled plots were grown with 8 cultivars (Table 1) known for carrying different *Lr* (leaf rust) resistance genes; this information having been obtained by multi-pathotype seedling tests based on a reference gene postulation method^30,31^. Two years were not included in the comparative analyses of populations collected during a given year as the number of collected isolates was too low to tackle our questions: (i) in 2007, 14 samples were collected on cultivar (cv.) Galibier and one on cv. Kalango; (ii) in 2009, 22 samples were collected on cv. Galibier and one on cv. Courtot. Similarly, two cultivars were not included in the analyses comparing populations between cultivars of origin due to the low number of samples: cv. Aubusson, with one sample collected in 2010, and cv. Renan with 9 samples collected in 2011. All methods, in particular collection of (cultivated) plant material, were carried out in accordance with relevant institutional, national, and international guidelines and legislation. Sampling of leaves from wheat volunteers was carried out with relevant permissions.

**Table 1:** Number of isolates collected from each cultivar during the 2007–2012 period.

Cultivar	*Lr* genes*	Year					
		2007	2008	2009	2010	2011	2012
Apache	*Lr13, Lr37*	0	17	0	29	23	33
Bologna	*Lr14a, Lr37*	0	28	0	0	25	8
Courtot	*Lr10, Lr14a*	0	18	1	7	10	0
Galibier	*Lr14a*	14	60	22	58	72	75
Ingenio	NA	0	10	0	0	0	7
Kalango	*Lr13, Lr14a*	1	10	0	10	10	13
Quality	no *Lr* gene	0	33	0	37	1	0
Renan	*Lr14a, Lr37*	0	0	0	0	9	0

*Major *Lr* (leaf rust resistance) genes conferring resistance to *P. triticina* isolates that do not possess virulence towards all the genes present in the cultivar.

Once collected, infected wheat leaves were placed in a duly identified envelope. Back in the lab, wheat leaf samples were recovered through inoculation on seedlings at the two-leaf stage of the susceptible wheat ‘Michigan Amber’. A single-uredinium isolate was produced from each of the collected leaves. Each isolate corresponded to one individual composing the pathogen population. The pathotype of each isolate was determined by inoculating a differential set of wheat lines according to standard techniques^23^. We used a set of 20 lines differing in the factors that determine their resistance to *Pt*: 17 Thatcher differential lines with different resistance genes to leaf rust (*Lr1, Lr2a, Lr2b, Lr2c, Lr3a, Lr3bg, Lr3ka, Lr10, Lr13, Lr14a, Lr15, Lr16, Lr17, Lr20, Lr23, Lr26, Lr37*), the Australian cultivar Harrier carrying *Lr17b*, and the susceptible control Morocco. The infection type on each of the differential lines was scored visually 10 days after inoculation according to the 0–4 scale described by Stakman et al.^32^. Two isolates are considered to belong to the same pathotype if they have the same combination of virulences. *Pt* samples collected during this study corresponded to 52 different pathotypes (Supplementary Material—Table S1).

Wheat field plots were georeferenced in QGIS^33^. The “distance matrix” tool was used to compute distances between all possible pairs of plots sampled during the same year (343 pairwise values in total). Maps were generated in R^34^ using the ‘rgdal’ package^35^. Pathogen population compositions in terms of pathotypes were compared between two plots using the Generalized Monte Carlo plug-in *test* with calibration (GMCPIC test^20^) adapted to low sample sizes. This test, implemented in the “StrainRanking” R package^36^, allowed us to infer similarity or difference in terms of identity and abundance of pathogen strains and to map the similarities in the geographic study area for getting a network vision of the population structure as conducted in Eck et al*.*’s paper^26^. We only retained in the analyses the tests applied to pairs of plots with sufficient sample sizes, namely each plot in the pair had to have at least 7 samples (n_min_ = 7). We calculated dissimilarity in strain composition for all pairwise combinations of populations (78 populations in total) so as to test whether strain composition is more similar between populations sampled in neighbouring plots and/or between plots cultivated with the same wheat cultivar through permutation tests. In these, the strain proportion vectors were randomly and uniformly reallocated to any sampled populations for any pair of plots. Each population collected in a given plot was characterised by strain diversity metrics, namely Shannon–Weaver diversity (H′), and Pielou’s evenness (J′) computed with the “vegan” R package^37^.

## Results

**Figure 1:**
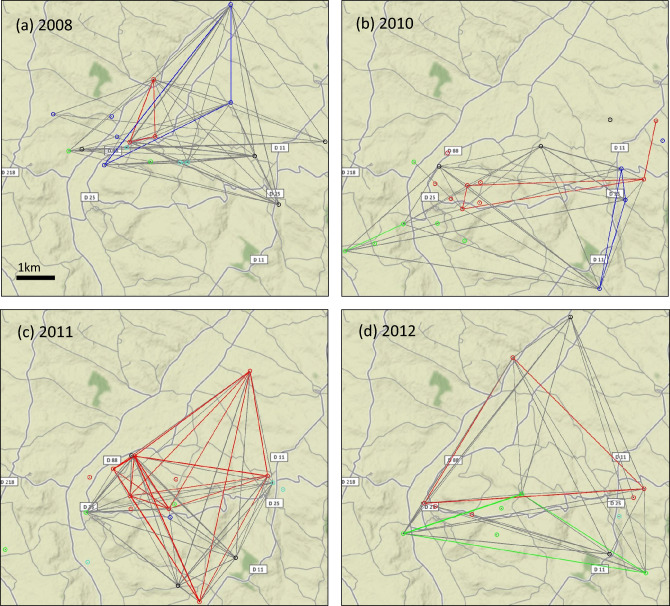
Spatio-temporal representation of the results of the similarity test of the pathotype composition of *P. triticina* populations (GMCPIC outputs) for different years. Pairs of plots that are linked by lines have similar composition of *P. triticina* populations according to the similarity test (with a significance level fixed at 0.05). Lack of a connecting line between two plots on the map indicates dissimilarity in the composition of pathogen populations. *P. triticina* populations, depicted as points that are coloured depending on the cultivar grown in each wheat field plot: red points correspond to plots grown with cv. Galibier, green points to cv. Apache, blue points to cv. Quality, turquoise points to cv. Bologna and black points to other less common cultivars in the landscape. Coloured lines link plots grown with the same cultivar; a colour indicating a given cultivar (same colour coding as for points). Grey lines link plots grown with different cultivars.

Pathogen strain identity and abundance were variable among the sampled field plots over the four years with both spatial and temporal variation in the strain composition. When representing the results of the similarity test of pathotype composition (Fig. 1), the links corresponding to plots with same cultivars are over-represented for each year of study, meaning that plots grown with same cultivars tend to have more similar pathogen compositions. The proportion of plot pairs with similar composition was variable among years from 49 to 89%. Based on permutation tests applied annually, the similarity of the cultivar among plots significantly corresponds to the similarity of pathogen compositions every year. In contrast, there is no significant effect of the distance on the similarity of pathogen compositions (Table 2).

**Table 2:** Effect of distance and of cultivar of origin on the difference in the pathotype composition of *P. triticina* population between two plots.

Year	Number of pairs of plots	Number of pairs of plots with similar composition (based on GMCPIC test)	Test statistic and *p*-value (permutation tests)	
			Distance effect	Cultivar effect
2008	119	59	t = 3.02	t = 8
			*p*-value = 0.15	*p*-value = 0.0001
2010	60	35	t = 3.48	t = 8
			*p*-value = 0.11	*p*-value = 0.0002
2011	66	59	t = 4.30	t = 19
			*p*-value = 0.60	*p*-value = 0.0022
2012	65	41	t = 5.80	t = 8
			*p*-value = 0.80	*p*-value = 0.0010

Differences between pathogen composition among populations were tested using the GMCPIC test (with a significance level fixed at 0.05) and the effect of distance and varietal identity on the similarity of compositions were tested using permutation tests. The test statistic (*t*) is the average distance among pairs of plots with similar composition for the test about the distance effect, and the number of pairs of plots with similar composition and similar cultivar for the test about the cultivar effect. The number of samples per plot was insufficient to carry out the analyses in 2009.

Differences between populations collected on different cultivars could be further investigated by disregarding spatial aspects (i.e. gathering isolates from plots grown with the same cultivars, for each year). Significant differences were observed but differed over time (Table 3). For example, the composition of pathogen populations on cv. Apache was similar to the one found on cv. Kalango in 2008 and 2012 but differed in 2010 and 2011. Diversity patterns of the overall pathogen populations based on cultivars of origin (isolates from plots of the same cultivars collected in different years were gathered) differed between cultivars, when considering richness and evenness. For example, *Pt* population sampled on cv. Quality (*n* = 83 samples) had both a high richness (H’ = 2.31) and a relatively high evenness (J’ = 0.77). This could be related to the absence of identified resistance genes in this cultivar. In contrast, cv. Galibier (*n* = 354 samples), which was the most frequent cultivar at the landscape scale during the study period, also had a high richness (H’ = 2.29) but a relatively low evenness (J’ = 0.62). This could be related to the presence of the *Lr14a* resistance gene in this cultivar for which we detected preferential cultivar-pathotype association due to a higher aggressiveness of the predominant pathotypes compared to other compatible pathotypes. Other cultivars such as cv. Apache, cv. Bologna and cv. Kalango all carry two *Lr* resistance genes and *Pt* populations sampled on these cultivars had a lower richness than cv. Galibier and cv. Quality.

**Table 3:** Diversity metrics and comparison of *P. triticina* populations from field plots of wheat volunteers grown with different wheat cultivars.

Cultivars	Apache	Bologna	Courtot	Galibier	Ingenio	Kalango	Quality	Renan
*Lr* genes*	*Lr13, Lr37*	*Lr14a, Lr37*	*Lr10, Lr14a*	*Lr14a*	NA	*Lr13, Lr14a*	no *Lr* gene	*Lr14a, Lr37*
2008	a	bd	bd	b	c	ac	e	–
2010	a	–	bc	b	–	c	d	–
2011	a	b	–	ab	–	c	–	d
2012	a	ac	–	ac	bc	ac	–	–
H’	1.92	1.93	1.46	2.29	1.78	1.58	2.31	1.75
J’	0.64	0.73	0.67	0.62	0.86	0.61	0.77	0.89
N	123	68	38	354	22	45	83	10

Different letters correspond to dissimilar *P. triticina* populations in terms of pathotype composition according to the similarity test conducted for each studied year. Abundance and diversity metrics are given over the entire study period for Shannon diversity (H’), Pielou evenness (J’), number of samples (N).

*Major *Lr* (leaf rust resistance) genes conferring resistance to *P. triticina* isolates that do not possess virulence towards all the genes present in the cultivar.

Regarding the impact of resistance genes present in cultivars on the composition of *Pt* populations, Table 4 shows the frequency of the main virulences and the presence of corresponding resistance genes in sampled cultivars. The *Lr14a* resistance gene was quite prevalent in the cultivars surveyed during this study (5 out of 8 cultivars). As expected, the corresponding virulence, which is necessary to infect a cultivar carrying this resistance, was present in almost all collected isolates. However, virulence towards *Lr10* was also highly present despite the absence of this resistance gene in most surveyed cultivars. Virulence towards *Lr13* and *Lr37* resistance genes strongly increased during the study period despite their limited presence in sampled cultivars. This suggests that despite their impact, the presence of resistance genes in local cultivars is not the only factor driving the local adaptation of *Pt* populations in terms of distribution of virulences.

**Table 4:** Number of isolates of *P. triticina* collected from surveyed wheat plots and identified by their virulence against four *Lr* resistance genes carried by grown cultivars in the study site for each sampling year.

Year	N	Proportion of samples with virulence towards *Lr* resistance gene* (%)					Proportion of samples from cultivar with *Lr* resistance gene* (%)				
		*Lr10*	*Lr13*	*Lr14a*	*Lr37*	*p*-value	*Lr10*	*Lr13*	*Lr14a*	*Lr37*	*p*-value
2008	176	99.4 aA	49.4 bA	99.4 a	51.1 bA	< 2.2 × 10^–16^	10.3 aA	15.3 aA	65.9 bA	25.6 cA	< 2.2 × 10^–16^
2010	142	95.8 aAB	78.2 bB	97.9 a	84.5 bB	2.4 × 10^–8^	4.9 aA	28.2 bBC	53.5 cB	21.1 bA	< 2.2 × 10^–16^
2011	160	92.5 aB	94.4 aC	100 b	96.3 aC	6.8 × 10^–3^	6.3 aA	20.6 bAC	78.7 cC	35.6 dBC	< 2.2 × 10^–16^
2012	154	94.2 aB	92.9 aC	99.4 b	92.9 aC	3.0 × 10^–2^	0.0 aB	29.9 bB	62.3 cA	26.6 dAC	< 2.2 × 10^–16^
*p*-value		1.5 × 10^–2^	< 2.2 × 10^–16^	0.2 (NS)	< 2.2 × 10^–16^		1.7 × 10^–3^	1.1 × 10^–3^	4.5 × 10^–07^	1.0 × 10^–2^	

N: total number of samples; lower case letters: differences within lines; upper case letters: differences within columns (khi2, R function “prop.test”, *p*-value < 0.05).

*Major *Lr* (leaf rust resistance) genes conferring resistance to *P. triticina* isolates that do not possess virulence towards all the genes present in the cultivar.

**Figure 2:**
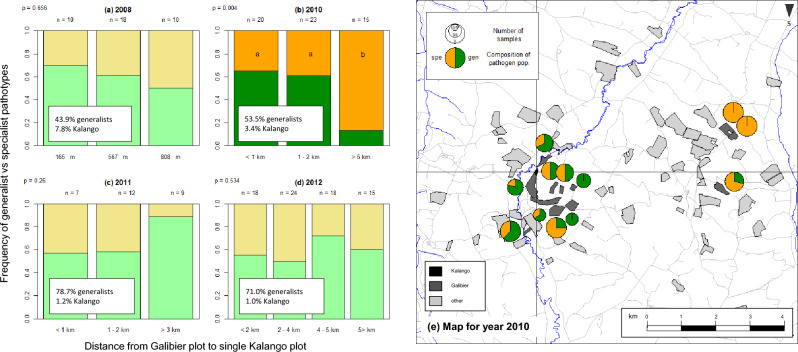
Variation of the composition of *P. triticina* populations between plots grown with the same cultivar at the landscape scale. The figures on the left (**a**–**d**) show the frequency of two categories of pathotypes (*Vir13* + *Vir14a* vs. *Vir14a* only) as a function of the distance between Galibier plots in which samples were collected and a Kalango plot which was located at the center of the sampling site (corresponding to the intersection of black lines on the map (**e**)). “*Virn*” stands for virulence towards the resistance gene “*Lrn*”.

Considering the most widely grown cultivar at the landscape scale (cv. Galibier), which was also the cultivar on which we found the highest number of wheat volunteers infected by *Pt*, spatio-temporal variation of pathogen composition in terms of virulence profile was observed (Fig. 2). Virulence towards *Lr14a* is necessary to infect both cv. Galibier and cv. Kalango, while virulence towards *Lr13* is facultative to infect cv. Galibier but necessary to infect cv. Kalango. As cv. Kalango was the only cultivar in this area combining both *Lr13* and *Lr14*, Kalango plots were expected to act as a local source of isolates combining virulence towards these two genes. The frequency of this category of isolates increased from 43.9% in 2008 to 71.0% in 2012 in the overall sampled population. The Kalango cultivar was quite rare in the study area with only one plot grown and sampled in the study site. At the regional scale, the frequency of cv. Kalango dropped from 7.8% in 2008 to 1% in 2012. In 2010, plots grown with cv. Galibier further than 5 km from the Kalango plot had significantly less isolates with both virulence *Lr13* and *Lr14a* than plots that were up to 2 km away from the Kalango plot (Fig. 2b,e).

## Discussion

Our study emphasised the high diversity of *Pt* populations on wheat volunteers at the landscape scale (52 pathotypes out of 642 leaf rust samples), in particular considering their virulence profiles to 18 *Lr* resistance genes (pathotype) and thus their ability to infect the wheat varieties grown in the study site. This is consistent with the diversity observed by Soubeyrand et al.^20^ in the same study site when considering sentinel plots of only two cultivars (extended in the present study to all wheat volunteer plots with 8 cultivars considered in total). Pathotype surveys, by monitoring pathogen populations in a given location or region, have emphasised the diversity found in space and time on different sources of resistance, in global^38,39^ and French^23,24^ *Pt* populations. This suggests that the inoculum provided by infected wheat volunteers is probably quite heterogeneous, both in time and space, depending on the deployment of within-crop diversity in agricultural landscapes. Studies of plant pathogen diversity conducted at the landscape scale remain rare^40,41^ as they require intensive field samplings and subsequent genotypic and/or phenotypic characterisation. Considering the landscape scale is particularly important to better understand the off-season period during which inoculum fluxes, beyond plot scale, are critical. This period remains a black box and is largely unexplored despite being recognized as a critical stage in pathogen metapopulation dynamics^4^. Indeed, ecological and evolutionary changes during the off-season play an important role in the dynamic behaviour of a wide range of other host–parasite systems^4,18,42^. In this study, we investigated how wheat volunteer patches constituting different host populations filtering pathogen populations by their *Lr* resistance genes, and distance between two wheat volunteer plots (dilution effect) could explain diversity (strain composition) in the *Pt* populations, surveyed within the 5-km radius zone.

The impact of cultivar on (dis)similarities of pathogen virulence profiles could be detected for all studied years (Table 2). The presence of some cultivars can considerably vary between years^43^, notably through regional ‘*boom-and-bust*’ cycles^4^ and associated changes in population genetics composition^3^. At the national scale (France), the most cultivated cultivars typically account for a high proportion of the wheat area^44^. In this study, cv. Galibier was highly present at the regional scale only. Interesting patterns could also be observed when investigating the overall impact of cultivar of origin ignoring spatial configuration, i.e. pooling samples from different field plots and, in some analyses, different years. Richness and evenness were quite different depending on the cultivar of origin (Table 3). The changes in prevalence of *Lr* resistance genes in the cultivars deployed in an agricultural area can be a strong factor impacting the level of diversity with host genotypes imposing selection on the corresponding pathogen populations, selecting for specifically adapted isolates to overcome its resistance^24^. We further investigated the variation of virulence patterns in the pathogen population compared to resistance patterns in deployed cultivars for different years of the study (Table 4). The 5-km radius study site was probably influenced by external inoculum pools, that were built-up by spores discharged from more or less distant wheat volunteer seedlings. This includes spores from neighbouring plots just outside the limit of the study site or from more distant plots at regional, national or even larger scales. Here, we discuss mainly national scale, for which relevant information (extensive *P. triticina* population survey describing annually its evolutionary dynamics in response to cultivar deployment) is available. Changes in pathotype distribution over the present study period highly related to the *Lr* genes prevalent at the national scale, when one pathotype, virulent against *Lr10, Lr13, Lr14a*, and *Lr37*, predominated in France from 2006 to 2016^24^. Thus, although most samples from the study were collected from cultivars that did not carry *Lr10*, *Vir10* was highly present in the local pathogen population (Table 1), in line with the composition of the national population. The diversity of pathogen populations detected locally in this study is particularly striking as, over the same period, a decrease in pathotype diversity was observed at national scale (from 2009 to 2014). Over the period of study, from 2007 to 2012, the predominant deployed *Lr* genes at national scale were *Lr13* and *Lr37* (more than 50% of the evaluated surfaces). This went hand in hand with a reduction of the use of *Lr10* (from 40% in 2007 to 23% in 2012) and an increase in the use of *Lr14a* (from 18% in 2007 to 37% in 2012). In that regard, the cultivars deployed in the local landscape of study are interesting because five out of the eight present in this case study carry the four *Lr* genes either solely or in combination (Table 1), along with the cv. Quality carrying no *Lr* gene, and thus expected to have a 'neutral' behaviour (no filter of *Lr* genes on this cultivar). These results suggest a combination of local and external selection pressures determining population composition at local scale. Cultivars grown locally could exert a selective pressure and filter isolates that have virulences required to infect them. If some *Lr* resistance genes are over-represented locally, they might impact the local pathogen population. However, the rareness of some resistance genes could have little impact. The presence of virulences that are unnecessary to infect local cultivars, could be determined by larger scale dynamics (e.g. national scale).

We detected no significant impact of distance between plots on pathotype compositions among *Pt* populations based on permutation tests (Table 2). This is paradoxical considering the large role of dispersal gradients in models simulating disease progression at landscape scale^45^. Experimental characterisation of dispersal gradients for fungal spores often involve inoculations with artificially high-density and localised inoculum^15,46–49^. But dispersal kernels of fungal spores have also been inferred at landscape scales embedding multiple and fragmented host populations^19,50^. The impact of distance might be detectable only if the population is heterogeneous enough (which depends on years as pathogen diversity is a result of strain assembly processes in their local environment conditions) and / or when there are enough isolates^20^. Despite the methodology developed by Soubeyrand et al.^20^, which was adapted to small samples (i.e. a few dozen isolates in the two samples to be compared), only part of the plots (with enough samples) could be included in the analysis. In this study, sample collection depended on the capacity to find wheat volunteers (which presence is strongly impacted by agricultural practices) with leaf rust symptoms. The sampling objective was at least 10 isolates but, this could not be accomplished for all plots, especially as some collected isolates could not be recovered in the lab due to viability loss. The prevalence of leaf rust on volunteers between successive wheat cropping seasons is dependent on a range of factors including (i) host population size^51^, (ii) connectivity between field plots^18,52^, (iii) favourable environmental conditions that do not exceed physiological tolerances (differential fitness and survival of strains), (iv) distance between spore sources, and (v) chance^53^ that will in turn drive pathogen populations diversity across landscapes, and determine how a subsequent epidemic will unfold. It is also dependent on the intensity of epidemics from the year before, that can explain fluctuations in sample sizes. For instance, the year 2007 was a high epidemic year in France (severe leaf rust disease outbreaks) and a high number of samples were collected on wheat volunteers the following fall in the study site (N = 159 samples in 2008). Furthermore, the method used to phenotype *Pt* samples is time-consuming (39 days to purify a sample, 16 days for pathotyping), with a need for dedicated facilities and skilled people. An annual sampling for this study at the landscape scale represents the same effective sampling effort as the French leaf rust national survey^23,24^. The development of new high-throughput molecular diagnostic tools, to directly detect in agricultural fields the presence of virulent isolates in populations without prior deep phenotyping (greenhouse pathogenicity tests), could help in further deciphering pathogen diversity across scales^54,55^. Moreover, citizen science surveillance involving farmers or technical advisory staff present in agricultural fields on a day-to-day basis would be very helpful to increase sampling coverage, and collect extensive data of presence and local abundance of *Pt* on wheat volunteers across spatial and temporal scales.

Despite the impossibility to explore systematically the interaction between spatial aspects and cultivar identity, mainly due to an insufficient number of samples, we were able to address this question for a particular case, considering the cv. Galibier, which was most present in the study area. We observed that the distance to a plot grown with a rare cultivar (Kalango) with a different resistance pattern (combination of *Lr13* and *Lr14a*) had an impact on virulence patterns found on the most widely grown cultivar Galibier (Fig. 2), but only for one year. This suggests that the mechanisms determining the pathotype composition of *Pt* populations on volunteer plots are probably quite complex. Indeed, factors other than distance between plots and cultivar identity play a role in shaping the diversity of pathogen populations such as local environmental conditions^56,57^ or demographic variation that impact the spatial distribution of strains in an area^58,59^. Additionally, factors impacting populations on wheat volunteers might be different from those impacting populations on cultivated hosts. Indeed, wheat volunteers are not expected to be exposed to fungicide treatments and could harbour more diverse pathogen populations. They could also cohabit with a diversity of weeds that constitute a wild compartment with more variable prevalence than cultivated canopies, as for virus infections in wild plant populations^60^. The ecology of agroecosystems frequently differs from natural ecosystems such that interactions that are common in managed systems may be rare in wild systems (e.g. case of viruses infected horticultural orchids^61^). Pathogen populations on wheat volunteers may play an important role, notably with respect to potential priority or founder effects^62,63^, during disease establishment with a small number of individuals that initiate a new population, and which have been highlighted as an important cause of differentiation among local populations^64^.

Our results suggest that volunteers acting as source of primary inoculum could play an important role in the adaptation thereof to local cultivars from one year to another. Location of newly sown plots in relation to former wheat plots with volunteers might have an impact on the composition of primary inoculum they receive. Distant inoculum could play a bigger role when small amounts of local primary inoculum are available from volunteers. The contribution balance probably depends on the year (years with more or less prevalence of wheat volunteers and/or leaf rust) and on a range of abiotic factors that remain to be better characterised (e.g. climatic conditions). Integrating multiple scales from plot to regional, national, and even continental scale seems useful to better understand these processes. Combining modelling with experimental studies including molecular tools, field and citizen science surveillance might also offer interesting perspectives to further investigate these aspects in particular to develop a full picture of how these patterns of pathogen diversity during the off-season link to variation in the incidence and severity of the onset of subsequent disease epidemics.

### Supplementary Information

Supplementary Tables.

## Data Availability

The data that support the findings of this study are available from the corresponding author upon reasonable request.
