# Supplementary material for: A landscape-scale field survey demonstrates the role of wheat volunteers as a local and diversified source of leaf rust inoculum
Source: Sci Rep. 2023 Nov 21;13:20411. doi: 10.1038/s41598-023-47499-6 (PMC10663564; doi:10.1038/s41598-023-47499-6)
Supplement: Supplementary file 1 — Supplementary Tables. [file 41598_2023_47499_MOESM1_ESM.pdf]

SUPPLEMENTARY MATERIAL

Wheat volunteers as a local and diversified source of leaf rust inoculum: a landscape-scale field survey. Boixel, A.-L.[1]; Goyeau, H.[1]; Berder, J. [1]; Moinard, J.[2]; Suffert, F.[1]; Soubeyrand, S.[3]; Sache, I.[4]; Vidal, T.[1*]

[1] Université Paris-Saclay, INRAE, AgroParisTech, UR BIOGER, 78850 Thiverval-Grignon, France
[2] DRAAF Midi-Pyrénées, 31074 Toulouse, France; [3] INRAE, BioSP, 84914 Avignon, France
[4] AgroParisTech, Ecole doctorale ABIES, Paris, France

Corresponding author: Tiphaine Vidal (tiphaine.vidal@inrae.fr)

TabS1.1 Virulence profiles of different pathotypes

Pathotype	N*	Virulence towards genes conferring resistance to *Puccinia triticina*				Virulence towards local cultivars				
		Lr10	*Lr13*	*Lr14a*	*Lr37*	Galibier	Kalango	Apache	Courtot	Aubusson
106314	288	1	1	1	1	1	1	1	1	1
073100	121	1	0	1	0	1	0	0	1	0
166317	38	1	1	1	1	1	1	1	1	1
165315	23	0	1	1	1	1	0	1	1	0
126377	16	1	1	1	1	1	1	1	1	1
167317	16	1	1	1	1	1	1	1	1	1
067317	15	1	1	1	1	1	1	1	1	1
106315	12	1	1	1	1	1	1	1	1	1
077317	11	1	1	1	1	1	1	1	1	1
166377	8	1	1	1	1	1	1	1	1	1
166316	7	1	1	1	1	1	1	1	1	1
063124	6	1	0	1	1	1	0	0	1	0
126376	6	1	1	1	1	1	1	1	1	1
002164	5	1	0	1	1	1	0	0	1	0
067337	5	1	1	1	1	1	1	1	1	1
006105	4	1	1	1	1	1	1	1	1	1
006106	4	1	1	1	1	1	1	1	1	1
165335	4	0	1	1	1	1	0	1	1	0
167316	4	1	1	1	1	1	1	1	1	1
167337	4	1	1	1	1	1	1	1	1	1
012160	3	1	0	1	0	1	0	0	1	0
106334	3	1	1	1	1	1	1	1	1	1
126374	3	1	1	1	1	1	1	1	1	1
002120	2	1	0	1	0	1	0	0	1	0
006104	2	1	1	1	1	1	1	1	1	1
012126	2	1	0	1	1	1	0	0	1	0
012162	2	1	0	1	0	1	0	0	1	0
033100	2	1	0	1	0	1	0	0	1	0
067316	2	1	1	1	1	1	1	1	1	1
166314	2	1	1	1	1	1	1	1	1	1

*N : number of samples from each pathotype included in the study

TabS1.2 Virulence profiles of different pathotypes (continued)

Pathotype	N	Virulence towards genes conferring resistance to *Puccinia triticina*				Virulence towards local cultivars				
		Lr10	***Lr13***	***Lr14a***	***Lr37***	**Galibier**	**Kalango**	**Apache**	**Courtot**	**Aubusson**
002002	1	1	0	0	0	0	0	0	0	0
010122	1	0	0	1	0	1	0	0	1	0
012002	1	1	0	0	0	0	0	0	0	0
012060	1	1	0	0	0	0	0	0	0	0
012104	1	1	0	1	1	1	0	0	1	0
067377	1	1	1	1	1	1	1	1	1	1
073120	1	1	0	1	0	1	0	0	1	0
077337	1	1	1	1	1	1	1	1	1	1
106217	1	1	1	0	1	0	1	1	0	1
106236	1	1	1	0	1	0	1	1	0	1
106316	1	1	1	1	1	1	1	1	1	1
106335	1	1	1	1	1	1	1	1	1	1
106336	1	1	1	1	1	1	1	1	1	1
165317	1	0	1	1	1	1	0	1	1	0
165334	1	0	1	1	1	1	0	1	1	0
165336	1	0	1	1	1	1	0	1	1	0
166315	1	1	1	1	1	1	1	1	1	1
166336	1	1	1	1	1	1	1	1	1	1
166337	1	1	1	1	1	1	1	1	1	1
166357	1	1	1	1	1	1	1	1	1	1
167336	1	1	1	1	1	1	1	1	1	1
173167	1	1	0	1	1	1	0	0	1	0

*N : number of samples from each pathotype included in the study
